# Extremal functions for Trudinger–Moser inequalities with nonnegative weights

**DOI:** 10.1186/s13660-018-1718-7

**Published:** 2018-05-25

**Authors:** Songbo Hou

**Affiliations:** Department of Applied Mathematics, College of Science, China Agricultural University, Beijing, P.R. China

**Keywords:** 46E35, Trudinger–Moser inequality, Extremal function, Blow-up analysis

## Abstract

Using blow-up analysis, the author proves the existence of extremal functions for Trudinger–Moser inequalities with nonnegative weights on bounded Euclidean domains or compact Riemannian surfaces. This extends recent results of Yang (J. Differ. Equ. 258:3161–3193, 2015) and Yang–Zhu (Proc. Am. Math. Soc. 145:3953–3959, 2017).

## Introduction

Let Ω be a smooth bounded domain in $\mathbb{R}^{2}$, $W^{1,p}(\Omega )$ be the usual Sobolev space and $W_{0}^{1,p}(\Omega )$ be the closure of $C_{0}^{\infty }(\Omega )$ in $W^{1,p}(\Omega )$. For $1\leq p<2$, the classical Sobolev theorem says that

$$\begin{aligned} W_{0}^{1,p}(\Omega )\hookrightarrow L^{q}(\Omega )\quad \text{for any } 1< q\leq2p/(2-p). \end{aligned}$$

As the limit case of the Sobolev inequality, the famous Trudinger–Moser inequality [3, 4] states

1.1 $$ \sup_{u\in W^{1,2}_{0} (\Omega ), \Vert \nabla u \Vert _{2}\leq1} \int_{\Omega }e^{4\pi u^{2}}\,dx < \infty . $$

This inequality is sharp in the sense that, for any $p>4\pi$, there exists a sequence $\{u_{j}\}\subset W_{0}^{1,2}(\Omega )$ with $\Vert \nabla u_{j} \Vert _{2}= 1$ such that $\int_{\Omega } e^{p u_{j}^{2}}\,dx\rightarrow \infty $ as $j\rightarrow\infty$. Furthermore, let $\{ u_{k}\}$ be a sequence of function in $W^{1,2}_{0}(\Omega )$ with $\Vert \nabla u_{k} \Vert _{2}=1$ such that $u_{k}\rightharpoonup u$ weakly in $W^{1,2}_{0}(\Omega )$. Lions [5] proved that, for any $p<1/(1- \Vert \nabla u \Vert ^{2}_{2})$, we have

1.2 $$ \limsup_{k\rightarrow \infty } \int_{\Omega }e^{4\pi pu_{k}^{2}}\,dx< \infty . $$

If $u\not\equiv0$, the inequality (1.2) gives more information than the Trudinger–Moser inequality (1.1). If $u\equiv0$, (1.2) is a consequence of (1.1). Motivated by this, Adimurthi and Druet [6] proved that, for any *α*, $0\leq \alpha <\lambda _{1}(\Omega )$,

1.3 $$ \sup_{u\in W^{1,2}_{0} (\Omega ), \Vert \nabla u \Vert _{2}\leq1} \int_{\Omega }e^{4\pi u^{2}(1+\alpha \Vert u \Vert ^{2}_{2})}\,dx < \infty , $$

where $\lambda _{1}({\Omega })$ is the first eigenvalue of the Laplace operator with respect to Dirichlet boundary condition. If $\alpha \geq \lambda _{1}(\Omega )$, then the supremum in (1.3) is infinity. The inequality (1.3) provides valuable supplementary information on (1.2). Note that if $\alpha =0$, (1.3) becomes the classical Trudinger–Moser inequality. Adimurthi and Druet’s result was extended by Yang to high dimensions [7] and compact Riemannian surfaces [8], and by Tintarev to a stronger version [9].

Denote

1.4 $$ \Vert u \Vert _{1,\alpha }= \biggl( \int_{\Omega } \vert \nabla u \vert ^{2}\,dx-\alpha \int_{\Omega }u^{2}\,dx \biggr)^{1/2} $$

for any $u\in W^{1,2}_{0}(\Omega )$ with $\int_{\Omega } \vert \nabla u \vert ^{2}\,dx-\alpha \int _{\Omega }u^{2}\,dx\geq0$. In [1], Yang proved that, for any *α*, $0\leq \alpha <\lambda _{1}(\Omega )$, we have

1.5 $$ \sup_{u\in W^{1,2}_{0}(\Omega ), \Vert u \Vert _{1,\alpha }\leq1} \int_{\Omega }e^{4\pi u^{2}}\,dx< \infty $$

and the supremum can be attained by some function $u_{0}\in W^{1,2}_{0}(\Omega )\cap C^{1}(\overline{\Omega })$ with $\Vert u_{0} \Vert _{1,\alpha }=1$. Let $\lambda _{1}(\Omega )<\lambda _{2}(\Omega )<\cdots$ be all distinct eigenvalues of the Laplace operator with respect to Dirichlet boundary condition and $E_{\lambda _{j}(\Omega )}$ be the eigenfunction space associated to $\lambda _{j}(\Omega )$. Noting that $W^{1,2}_{0}(\Omega )$ is a Hilbert space, for any positive integer *l*, we have

$$\begin{aligned} W^{1,2}_{0}(\Omega )=E_{l}\oplus E_{l}^{\perp}, \end{aligned}$$

where

1.6 $$ E_{l}=E_{\lambda _{1}(\Omega )}\oplus E_{\lambda _{2}(\Omega )}\oplus\cdots\oplus E_{\lambda _{l}(\Omega )} $$

and

1.7 $$ E_{l}^{\perp}= \biggl\{ u\in W^{1,2}_{0}( \Omega ): \int_{\Omega }uv\,dx=0, \forall v\in E_{l} \biggr\} . $$

It was also proved by Yang [1] that, for any *α*, $0\leq \alpha <\lambda _{l+1}(\Omega )$, we have

1.8 $$ \sup_{u\in E_{l}^{\perp}, \Vert u \Vert _{1,\alpha }\leq1} \int_{\Omega }e^{4\pi u^{2}}\,dx< \infty $$

and the supremum can be attained by some $u_{0}\in E_{l}^{\perp}\cap C^{1}(\overline{\Omega })$ with $\Vert u_{0} \Vert _{1,\alpha }=1$. The analogs of (1.5) and (1.8) still hold on compact Riemannian surfaces.

Our first result is the following.

### Theorem 1

*Let* Ω *be a smooth bounded domain in* $\mathbb{R}^{2}$, $\lambda _{1}(\Omega )$ *be the first eigenvalue of the Laplace operator with Dirichlet boundary condition*, *and* *h* *be in* $C^{0}(\overline{\Omega })$ *with* $h\geq0$ *and* $h\not\equiv0$. *Then we have*, *for any* $0\leq \alpha <\lambda _{1}(\Omega )$, *the supremum*

$$\begin{aligned} \sup_{u\in W^{1,2}_{0}(\Omega ), \Vert u \Vert _{1, \alpha }\leq1 } \int_{\Omega } he^{4\pi u^{2}}\,dx \end{aligned}$$

*can be attained by some* $u_{0}\in W^{1,2}_{0}(\Omega ) \cap C^{1}(\overline{\Omega })$ *satisfying* $\Vert u_{0} \Vert _{1,\alpha }=1$, *where* $\Vert \cdot \Vert _{1,\alpha }$ *is defined as in* (1.4).

When the high order eigenvalues are involved, we have a similar result.

### Theorem 2

*Let* Ω *be a smooth bounded domain in* $\mathbb{R}^{2}$, $\lambda _{l+1}(\Omega )$ *be the* $(l+1)$*th eigenvalue of the Laplace operator with Dirichlet boundary condition*, *and* *h* *be in* $C^{0}(\overline{\Omega })$ *with* $h\geq0$ *and* $h\not \equiv0$. *Then we see that*, *for any* $0\leq \alpha <\lambda _{l+1}(\Omega )$, *the supremum*

$$\begin{aligned} \sup_{u\in E_{l}^{\perp}, \Vert u \Vert _{1, \alpha }\leq1 } \int _{\Omega } he^{4\pi u^{2}}\,dx \end{aligned}$$

*can be attained by some* $u_{0}\in E_{l}^{\perp}\cap C^{1}(\overline {\Omega })$ *satisfying* $\Vert u_{0} \Vert _{1,\alpha }=1$, *where* $E_{l}^{\perp}$ *is defined as in* (1.7) *and* $\Vert \cdot \Vert _{1,\alpha }$ *defined as in* (1.4).

Similar results hold on compact Riemannian surfaces. Denote by $(\Sigma , g)$ a compact Riemannian surface without boundary, by $\nabla _{g}$ its gradient operator and by $\Delta _{g}$ the Laplace–Beltrami operator, respectively. Let $\lambda _{1}(\Sigma)$ be the first eigenvalue of $\Delta _{g}$. Denote

1.9 $$ \Vert u \Vert _{1,\alpha }= \biggl( \int_{\Sigma} \vert \nabla _{g} u \vert ^{2} \,dx-\alpha \int_{\Sigma }u^{2}\,dv_{g} \biggr)^{1/2} $$

for all $u\in W^{1,2}(\Sigma )$ with $\int_{\Sigma} \vert \nabla _{g} u \vert ^{2}\,dx-\alpha \int _{\Sigma}u^{2}\,dv_{g}\geq0$. Then we have the following theorem.

### Theorem 3

*Let* $(\Sigma ,g)$ *be a compact Riemannian surface without boundary*, *h* *be in* $C^{0}(\Sigma )$ *with* $h\geq0$ *and* $h\not\equiv0$. *Then*, *for any* *α*, $0\leq \alpha <\lambda _{1}(\Sigma )$, *the supremum*

$$\begin{aligned} \sup_{u\in W^{1,2}(\Sigma ),\int_{\Sigma }u \,dv_{g}=0, \Vert u \Vert _{1,\alpha }\leq 1} \int _{\Sigma }he^{4\pi u^{2}}\,dv_{g} \end{aligned}$$

*can be attained by some* $u_{0}\in W^{1,2}(\Sigma )\cap C^{1}{(\Sigma ) }$ *satisfying* $\Vert u_{0} \Vert _{1,\alpha }=1$ *and* $\int_{\Sigma }u_{0}\,dv_{g}=0$.

### Corollary 4

*Let* $(\Sigma ,g)$ *be a compact Riemannian surface without boundary*, *h* *be in* $C^{0}(\Sigma )$ *with* $h\geq0$ *and* $h\not\equiv0$. *For any* *α*, $0\leq \alpha <\lambda _{1}(\Sigma )$, $\forall u\in W^{1,2}(\Sigma )\textit{ with }\int_{\Sigma}u \,dv_{g}=0$, *define*

$$\begin{aligned} J(u)=\frac{1}{2} \biggl( \int_{\Sigma} \vert \nabla _{g} u \vert ^{2} \,dv_{g}-\alpha \int _{\Sigma}u^{2}\,dv_{g} \biggr)-8\pi\log \int_{\Sigma} he^{u} \,dv_{g}. \end{aligned}$$

*Then we have the weak Trudinger–Moser inequality* $J(u)\geq-C$, *where* *C* *is a positive constant depending only on* $(\Sigma,g)$ *and* *α*.

If *h* is strictly positive and $J(u)$ has no minimizer on $\mathcal {H}=\{ u\in W^{1,2}(\Sigma ): \int_{\Sigma}u\,dv_{g}=0\}$, Yang and Zhu [10] calculated the infimum of $J(u)$ on $\mathcal{H}$ by using the method of blow-up analysis. One may refer to [11] for earlier results on the functional

$$\frac{1}{2} \int_{\Sigma} \vert \nabla _{g} u \vert ^{2} \,dv_{g}+8\pi \int_{\Sigma }u\,dv_{g}-8\pi\log \int_{\Sigma}he^{u} \,dv_{g}. $$

Let $\lambda _{1}(\Sigma )<\lambda _{2}(\Sigma )<\cdots$ be all distinct eigenvalues of $\Delta _{g}$ and $E_{\lambda _{i}(\Sigma )}$ be the eigenfunction space associated to $\lambda _{i}(\Sigma )$. For any positive integer *l*, denote

$$\begin{aligned} E_{l}=E_{\lambda _{1}(\Sigma )}\oplus E_{\lambda _{2}(\Sigma )}\oplus\cdots \oplus E_{\lambda _{l}(\Sigma )} \end{aligned}$$

and

$$\begin{aligned} E_{l}^{\perp}= \biggl\{ u\in W^{1,2}(\Sigma ): \int_{\Sigma }uv\,dv_{g}=0, \forall v\in E_{l} \biggr\} . \end{aligned}$$

Similar to Theorem 2, we obtain the following.

### Theorem 5

*Let* $(\Sigma ,g)$ *be a compact Riemannian surface without boundary*, *h* *be in* $C^{0}(\Sigma )$ *with* $h\geq0$ *and* $h\not\equiv0$. *Then*, *for any* *α*, $0\leq \alpha <\lambda _{l+1}(\Sigma )$, *the supremum*

$$\begin{aligned} \sup_{u\in E_{l}^{\perp}, \int_{\Sigma}u\,dv_{g}=0, \Vert u \Vert _{1,\alpha }\leq 1} \int_{\Sigma }he^{4\pi u^{2}}\,dv_{g} \end{aligned}$$

*can be attained by some* $u_{0}\in E_{l}^{\perp}\cap C^{1}{(\Sigma ) }$ *satisfying* $\Vert u_{0} \Vert _{1,\alpha }=1$ *and* $\int_{\Sigma }u_{0}\,dv_{g}=0$.

Existence of extremal functions for Trudinger–Moser inequality can be traced back to Carleson and Chang [12], where the unit ball case was treated. Later contributions in this direction include M. Struwe [13], Flucher [14], Lin [15], Ding–Jost–Li–Wang [11], Adimurthi–Struwe [16], Li [17], Adimurthi–Druet [6], and so on. In our proof, we use the blow-up method. Compared with [1], there are some different key points. First, we derive the different Euler–Lagrange equation on which the analysis is performed. Then we prove that *h* must be positive at the blow-up point. Hence we use the different scaling when define the maximizing sequences of functions. We also obtain the different upper bound of the subcritical functionals. Finally, when proving the existence of the extremal function, we obtain the different lower bounds for the integrals of test functions constructed in Sects. 2–5. It should be remarked that our analysis on the weight *h* is essentially different from that of Yang and Zhu [2], where a weak version of Trudinger–Moser inequality was studied.

The rest of the paper is arranged as follows. In Sects. 2 and 3, we prove the main results in the Euclidean case (Theorems 1 and 2). In Sects. 4 and 5, we prove the main results in the Riemannian surface case (Theorems 3 and 5).

## Proof of Theorem 1

### The subcritical functionals

In this subsection, using the method in the calculus of variations, we prove the existence of maximizers for the subcritical functionals.

#### Lemma 6

*For any* $0<\epsilon <4\pi$, *any* $0\leq \alpha <\lambda _{1}(\Omega )$, *there exists some* $u_{\epsilon }\in W^{1,2}_{0}(\Omega )\cap C^{1}(\overline{\Omega })$ *with* $\Vert u_{\epsilon }\Vert _{1,\alpha }=1$ *such that*

2.1 $$ \int_{\Omega }h e^{(4\pi-\epsilon )u_{\epsilon }^{2}}\,dx=\sup_{u\in W^{1,2}_{0}(\Omega ), \Vert u \Vert _{1, \alpha }\leq1 } \int_{\Omega } he^{(4\pi-\epsilon )u^{2}}\,dx, $$

*where* $\Vert \cdot \Vert _{1,\alpha }$ *is defined as in* (1.4).

#### Proof

For $0<\epsilon <4\pi$, we choose a function sequence $u_{j}\in W^{1,2}_{0}(\Omega )$ such that

$$\begin{aligned} \int_{\Omega } \vert \nabla u_{j} \vert ^{2} \,dx-\alpha \int_{\Omega }u_{j}^{2}\,dx\leq1 \end{aligned}$$

and

2.2 $$ \int_{\Omega }h e^{(4\pi-\epsilon )u_{j}^{2}}\,dx\rightarrow\sup _{u\in W^{1,2}_{0}(\Omega ), \Vert u \Vert _{1,\alpha }\leq1 } \int_{\Omega } he^{(4\pi-\epsilon )u^{2}}\,dx $$

as $j\rightarrow\infty$. Then there exists some $u_{\epsilon }\in W^{1,2}_{0}(\Omega )$ such that up to a subsequence,

$$\begin{aligned} &u_{j}\rightharpoonup u_{\epsilon } \quad\text{weakly in } W^{1,2}_{0}(\Omega ), \\ &u_{j}\rightarrow u_{\epsilon } \quad\text{strongly in } L^{p}(\Omega ), \forall p\geq1, \\ & u_{j}\rightarrow u_{\epsilon } \quad\text{a.e. in } \Omega . \end{aligned}$$

Using a similar argument in the spirit of the one in [1], we find that $he^{(4\pi-\epsilon )u_{j}^{2}}$ is bounded in $L^{q}(\Omega )$ for some $q>1$. Then we get $he^{(4\pi-\epsilon )u_{j}^{2}}\rightarrow he^{(4\pi-\epsilon )u_{\epsilon }^{2}}$ strongly in $L^{1}(\Omega )$. This together with (2.2) immediately yields (2.1). We claim that $\Vert u_{\epsilon }\Vert _{1,\alpha }=1$. Otherwise $\Vert u_{\epsilon }\Vert _{1,\alpha } <1$. It follows that

2.3 $$\begin{aligned} \sup_{u\in W^{1,2}_{0}(\Omega ), \Vert u \Vert _{1,\alpha }\leq1 } \int _{\Omega } h e^{(4\pi-\epsilon )u^{2}}\,dx&\geq \int_{\Omega } h e^{(4\pi-\epsilon )\frac{u_{\epsilon }^{2}}{ \Vert u_{\epsilon }\Vert ^{2}_{1,\alpha }}}\,dx \\ & > \int_{\Omega } h e^{(4\pi-\epsilon )u_{\epsilon }^{2}}\,dx. \end{aligned}$$

There is a contradiction between in (2.1) and (2.3). Hence $\Vert u_{\epsilon }\Vert _{1,\alpha }=1$. □

Moreover, the Euler–Lagrange equation for $u_{\epsilon }$ is

2.4 $$ \textstyle\begin{cases}-\Delta u_{\epsilon}-\alpha u_{\epsilon }=\frac{1}{\lambda _{\epsilon}}hu_{\epsilon}e^{(4\pi-\epsilon ) u_{\epsilon}^{2}} &\text{in } \Omega ,\\ u_{\epsilon }>0 &\text{in } \Omega ,\\ \lambda_{\epsilon}=\int_{\Omega } h u_{\epsilon}^{2}e^{(4\pi-\epsilon) u_{\epsilon}^{2}}\,dx. \end{cases} $$

Using elliptic estimates, we get $u_{\epsilon }\in C^{1}(\overline{\Omega })$. Let $c_{\epsilon }=u_{\epsilon }(x_{\epsilon })=\max_{\Omega }u_{\epsilon }$. If $c_{\epsilon }$ is bounded, the existence of the extremal function is trivial by standard elliptic estimates. Thus we assume that $c_{\epsilon }\rightarrow\infty$ and $x_{\epsilon }\rightarrow x_{0}\in \overline{\Omega }$. A result of Gidas, Ni and Nirenberg on page 223 of [18] implies $x_{0}\notin\partial \Omega $.

Using the same argument as the one in step 2 of [1], we get the energy concentration. For the function sequence $u_{\epsilon }$, we have $u_{\epsilon }\rightharpoonup0$ weakly in $W^{1,2}_{0}(\Omega )$, $u_{\epsilon }\rightarrow0$ strongly in $L^{q}(\Omega )$ for any $q>1$, and $\vert \nabla u_{\epsilon }\vert ^{2}\,dx\rightharpoonup\delta_{x_{0}}$ in the sense of measure as $\epsilon \rightarrow0$, where $\delta_{x_{0}}$ denotes the Dirac measure centered at $x_{0}$.

Next we prove that *h* is positive at the blow-up point $x_{0}$. This property plays an important part in our analysis.

#### Lemma 7

*There holds* $h(x_{0})>0$.

#### Proof

We prove it by contradiction. Suppose that $h(x_{0})=0$. Note that up to a sequence

$$\begin{aligned} \lim_{\epsilon \rightarrow0} \int_{\Omega } h \bigl(e^{(4\pi-\epsilon )u_{\epsilon }^{2}}-1 \bigr)\,dx=\sup _{u\in W^{1,2}_{0}(\Omega ), \Vert u \Vert _{1, \alpha }\leq1 } \int_{\Omega } h \bigl(e^{4\pi u^{2}}-1 \bigr)\,dx\geq\eta, \end{aligned}$$

where *η* is a positive constant. Let *ϵ* be sufficiently small such that

2.5 $$ \int_{\Omega } h \bigl(e^{(4\pi-\epsilon )u_{\epsilon }^{2}}-1 \bigr)\,dx> \frac{\eta}{2}. $$

Choose $r>0$ such that $B_{r}(x_{0})\subset \Omega $. Then

2.6 $$\begin{aligned} &\int_{\Omega } h \bigl(e^{(4\pi-\epsilon )u_{\epsilon }^{2}}-1 \bigr)\,dx \\ &\quad= \int _{B_{r}(x_{0})}h \bigl(e^{(4\pi-\epsilon )u_{\epsilon }^{2}}-1 \bigr)\,dx+ \int_{\Omega \setminus B_{r}(x_{0})}h \bigl(e^{(4\pi-\epsilon )u_{\epsilon }^{2}}-1 \bigr)\,dx \\ &\quad= o_{r}(1) \int_{B_{r}(x_{0})} \bigl(e^{(4\pi-\epsilon )u_{\epsilon }^{2}}-1 \bigr)\,dx+ \int _{\Omega \setminus B_{r}(x_{0})}h \bigl(e^{(4\pi-\epsilon )u_{\epsilon }^{2}}-1 \bigr)\,dx, \end{aligned}$$

where $o_{r}(1)\rightarrow0$ as $r\rightarrow0$.

Choose *r* sufficiently small such that

2.7 $$ o_{r}(1) \int_{B_{r}(x_{0})} \bigl(e^{(4\pi-\epsilon )u_{\epsilon }^{2}}-1 \bigr)\,dx\leq o_{r}(1) \int_{\Omega } \bigl(e^{4\pi u_{\epsilon }^{2}}-1 \bigr)\,dx\leq \frac {\eta}{4}. $$

Here we have used the Trudinger–Moser inequality (1.5).

Applying elliptic estimates to the Euler–Lagrange equation (2.4), we obtain $u_{\epsilon }\rightarrow0$ in $C^{1}_{\mathrm{loc}}(\Omega \setminus \{x_{0}\})$. Hence

2.8 $$ \int_{\Omega \setminus B_{r}(x_{0})}h \bigl(e^{(4\pi-\epsilon )u_{\epsilon }^{2}}-1 \bigr)\,dx=o(\epsilon ). $$

Combining (2.6), (2.7) and (2.8), we find that if *ϵ* is sufficiently small,

2.9 $$ \int_{\Omega } h \bigl(e^{(4\pi-\epsilon )u_{\epsilon }^{2}}-1 \bigr)\,dx< \frac{\eta}{2}. $$

There is a contradiction between (2.5) and (2.9). Hence $h(x_{0})>0$. □

### Blow-up analysis

We shall analyze the behavior of the maximizers by using a blow-up analysis. Let

$$\begin{aligned} r_{\epsilon }=\sqrt{ \lambda _{\epsilon }}\bigl[h(x_{0}) \bigr]^{-1/2}c_{\epsilon }^{-1}e^{-(2\pi-\epsilon /2)c_{\epsilon }^{2}}. \end{aligned}$$

Using the Hölder inequality and the classical Trudinger–Moser inequality, we have

$$\begin{aligned} \int_{\Omega }hu_{\epsilon }^{2}e^{(4\pi-\epsilon )u_{\epsilon }^{2}}\,dx \leq e^{\delta c_{\epsilon }^{2}} \int_{\Omega }h u_{\epsilon }^{2}e^{(4\pi-\epsilon -\delta)u_{\epsilon }^{2}}\,dx \leq Ce^{\delta c_{\epsilon }^{2}}, \end{aligned}$$

where $0<\delta<4\pi$, *C* depends only on *h* and *δ*. Thus we get

$$r^{2}_{\epsilon }\leq C \bigl[h(x_{0}) \bigr]^{-1}c_{\epsilon }^{-2}e^{-(4\pi-\epsilon -\delta)c_{\epsilon }^{2}}\rightarrow0 $$

as $\epsilon \rightarrow0$.

Set

$$\Omega _{\epsilon }=\bigl\{ x\in\mathbb{R}^{2}:x_{\epsilon }+r_{\epsilon }x\in \Omega \bigr\} . $$

We define two sequences of functions on $\Omega _{\epsilon }$:

$$\begin{aligned} \psi_{\epsilon }(x)=c_{\epsilon }^{-1}u_{\epsilon }(x_{\epsilon }+r_{\epsilon }x), \qquad\varphi_{\epsilon }(x)=c_{\epsilon }\bigl(u_{\epsilon }(x_{\epsilon }+r_{\epsilon }x)-c_{\epsilon }\bigr). \end{aligned}$$

They satisfy the following equation:

$$\begin{aligned} &{-}\Delta \psi_{\epsilon }=\alpha r_{\epsilon }^{2} \psi_{\epsilon }+\frac{\psi_{\epsilon }h e^{(4\pi-\epsilon )(u_{\epsilon }^{2}-c_{\epsilon }^{2})}}{c_{\epsilon }^{2} h(x_{0})} \quad\text{in } \Omega _{\epsilon }, \\ &{-}\Delta \varphi_{\epsilon }=\alpha r_{\epsilon }^{2} c_{\epsilon }^{2}\psi_{\epsilon }+\frac{\psi_{\epsilon }h e^{(4\pi -\epsilon )(1+\psi_{\epsilon })\varphi_{\epsilon }}}{h(x_{0})}\quad \text{in } \Omega _{\epsilon }. \end{aligned}$$

It is clear that $\Omega _{\epsilon }\rightarrow\mathbb{R}^{2}$ as $\epsilon \rightarrow0$. Noting that $\vert \psi_{\epsilon }\vert \leq1$ and $\Delta \psi_{\epsilon }\rightarrow0$ uniformly in $\Omega _{\epsilon }$ as $\epsilon \rightarrow0$ and using the elliptic estimates, we get $\psi_{\epsilon }\rightarrow\psi$ in $C_{\mathrm{loc}}^{1}(\mathbb{R}^{2})$, where *ψ* is a bounded harmonic function in $\mathbb{R}^{2}$. Since $\psi(0)=\lim_{\epsilon \rightarrow 0}\psi_{\epsilon }(0)=1$, we have by the Liouville theorem

$$\begin{aligned} \psi_{\epsilon }\rightarrow1\quad \text{in } C_{{\mathrm{loc}}}^{1} \bigl(\mathbb {R}^{2} \bigr). \end{aligned}$$

Similarly, we have by the elliptic estimates

$$\begin{aligned} \varphi_{\epsilon }\rightarrow\varphi\quad\text{in } C_{{\mathrm{loc}}}^{1} \bigl(\mathbb {R}^{2} \bigr), \end{aligned}$$

where *φ* satisfies

$$\begin{aligned} -\Delta \varphi=e^{8\pi\varphi}\quad \text{in } \mathbb{R}^{2} \end{aligned}$$

and

$$\begin{aligned} \varphi(0)=0. \end{aligned}$$

We calculate

$$\begin{aligned} \int_{\mathbb {B}_{R}(0)}e^{8\pi\varphi}\,dx&\leq \limsup _{\epsilon \rightarrow 0} \int _{\mathbb {B}_{R}(0)}e^{(4\pi-\epsilon )(1+\psi_{\epsilon })\varphi_{\epsilon }}\,dx \\ &\leq \limsup_{\epsilon \rightarrow0}\lambda _{\epsilon }^{-1} \int_{\mathbb {B}_{Rr_{\epsilon }}(x_{\epsilon })}h(x_{0})c_{\epsilon }^{2}e^{(4\pi-\epsilon )u_{\epsilon }^{2}(y)}\,dy \\ &\leq \limsup_{\epsilon \rightarrow0}\lambda _{\epsilon }^{-1} \int_{\mathbb {B}_{Rr_{\epsilon }}(x_{\epsilon })}h(y)u_{\epsilon }^{2}(y)e^{(4\pi-\epsilon )u_{\epsilon }^{2}(y)} \,dy \\ &\leq 1. \end{aligned}$$

A result of Chen and Li [19] implies that

$$\begin{aligned} \varphi(x)=-\frac{1}{4\pi}\log \bigl(1+\pi \vert x \vert ^{2} \bigr) \end{aligned}$$

and

$$\begin{aligned} \int_{\mathbb{R}^{2}} e^{8\pi\varphi}\,dx=1. \end{aligned}$$

For the convergence behavior away from $x_{0}$, we have $c_{\epsilon }u_{\epsilon }\rightharpoonup G$ weakly in $W^{1,p}_{0}(\Omega )$ for any $1< p<2$, strongly in $L^{q}(\Omega )$ for any $q\geq1$ and in $C_{{\mathrm{loc}}}^{1}(\overline{\Omega }\backslash\{x_{0}\})$, where *G* is a Green function satisfying

$$\begin{aligned} \textstyle\begin{cases}-\Delta G-\alpha G=\delta_{x_{0}}\quad \text{in } \Omega ,\\ G=0 \quad\text{on } \partial \Omega , \end{cases}\displaystyle \end{aligned}$$

where $\delta_{x_{0}}$ is the Dirac measure centered at $x_{0}$.

*G* can be represented by

$$\begin{aligned} G=-\frac{1}{2\pi}\log \vert x-x_{0} \vert +A_{0}+ \Phi(x), \end{aligned}$$

where $A_{0}$ is a constant depending on $x_{0}$ and *α*, $\Phi\in C^{1}(\Omega )$ with $\Phi(x_{0})=0$.

### Upper bound estimates

Let *δ* be small such that $B_{\delta}(x_{0})\subset \Omega $. Let $s_{\epsilon }=\sup_{\partial B_{\delta}(x_{0})}u_{\epsilon }$ and $\bar{u}_{\epsilon }=(u_{\epsilon }-s_{\epsilon })^{+}$. Then $\bar{u}\in W_{0}^{1,2}(B_{\delta}(x_{0}))$. Let $\tau_{\epsilon }=1-\frac {1}{c_{\epsilon }^{2}} (\frac{1}{2\pi}\log\frac{1}{\delta}+A_{0}+o_{\delta}(1)+o_{\epsilon }(1) )$. Then, by the calculation in step 4 of Sect. 3 in [1], we get

2.10 $$ \limsup_{\epsilon \rightarrow0} \int_{B_{\delta}(x_{0})} \bigl(e^{4\pi\bar {u}_{\epsilon }^{2}/ \tau _{\epsilon }}-1 \bigr)\,dx\leq\pi \delta^{2} e $$

and

$$(4\pi-\epsilon )u_{\epsilon }^{2}\leq4\pi\bar{u}_{\epsilon }^{2}/ \tau_{\epsilon }-2\log\delta +4\pi A_{0}+o(1). $$

Hence

$$\begin{aligned} \int_{B_{Rr_{\epsilon }}(x_{\epsilon })}he^{(4\pi-\epsilon )u_{\epsilon }^{2}}\,dx&\leq \delta ^{-2}e^{4\pi A_{0}+o(1)} \int_{B_{Rr_{\epsilon }}(x_{\epsilon })}he^{4\pi\bar{u}_{\epsilon }^{2}/\tau _{\epsilon }}\,dx \\ &=\delta^{-2}e^{4\pi A_{0}+o(1)} \int_{B_{Rr_{\epsilon }}(x_{\epsilon })}h \bigl(e^{4\pi\bar {u}_{\epsilon }^{2}/\tau_{\epsilon }}-1 \bigr)\,dx+o(1) \\ &\leq \delta^{-2}e^{4\pi A_{0}+o(1)}h(x_{0}) \int_{B_{\delta}(x_{0})} \bigl(e^{4\pi\bar {u}_{\epsilon }^{2}/\tau_{\epsilon }}-1 \bigr)\,dx. \end{aligned}$$

This together with (2.10) leads to

$$\begin{aligned} \limsup_{\epsilon \rightarrow0} \int_{B_{Rr_{\epsilon }}(x_{\epsilon })}he^{(4\pi-\epsilon )u_{\epsilon }^{2}}\,dx\leq \pi h(x_{0})e^{1+4\pi A_{0}}. \end{aligned}$$

The argument in the proof of Lemma 3.3 in [20] yields

$$\begin{aligned} \lim_{\epsilon \rightarrow0} \int_{\Omega } he^{(4\pi-\epsilon )u_{\epsilon }^{2}}\,dx&\leq \gamma +\lim _{R\rightarrow +\infty}\limsup_{\epsilon \rightarrow0} \int_{B_{Rr_{\epsilon }}(x_{\epsilon })}he^{(4\pi-\epsilon )u_{\epsilon }^{2}}\,dx\\ &\leq\gamma+\pi h(x_{0})e^{1+4\pi A_{0}}, \end{aligned}$$

where $\gamma=\int_{\Omega }h \,dx$. This implies that

2.11 $$ \sup_{u\in W^{1,2}_{0}(\Omega ), \Vert u \Vert _{1, \alpha }\leq1 } \int_{\Omega } he^{4\pi u^{2}}\,dx\leq\gamma+\pi h(x_{0})e^{1+4\pi A_{0}}. $$

### Existence of extremal functions

Let $r(x)= \vert x-x_{0} \vert $. Define

2.12 $$ \phi_{\epsilon }(x)=\textstyle\begin{cases}c+\frac{1}{c} (-\frac{1}{4\pi}\log(1+\pi \frac {r^{2}}{\epsilon ^{2}})+B ) & \text{for } x\leq R\epsilon ,\\ \frac{G-\eta\Phi}{c}& \text{for } R\epsilon \leq r\leq2R\epsilon ,\\ \frac{G}{c}& \text{for } r>2R\epsilon , \end{cases} $$

as in [1], where *c* and *B* are constants, $R=-\log \epsilon $, $\eta\in C^{\infty}_{0}(B_{2R\epsilon }(x_{0}))$ with $\eta=1$ on $B_{R\epsilon }(x_{0})$ and $\Vert \nabla \eta \Vert _{L^{\infty}}=O(\frac{1}{R\epsilon })$. Choose

$$\begin{aligned} &c= \biggl(\frac{-\log \epsilon -2\pi B+2\pi A_{0}+\frac{1}{2}\log\pi+O (\frac {1}{R^{2}} )}{2\pi} \biggr)^{1/2}, \\ &B=\frac{1}{4\pi}+O \biggl( \frac{1}{R^{2}} \biggr) +O \bigl(R\epsilon \log(R\epsilon ) \bigr), \end{aligned}$$

as in [1] such that $\phi_{\epsilon }\in W^{1,2}_{0}(\Omega )$ and $\Vert \phi _{\epsilon }\Vert _{1,\alpha }=1$. Then we get

$$\begin{aligned} \int_{B_{R\epsilon }(x_{0})}he^{4\pi\phi_{\epsilon }^{2}}\,dx\geq\pi h(x_{0})e^{1+4\pi A_{0}}+O \biggl(\frac{1}{R^{2}} \biggr) \end{aligned}$$

and

$$\begin{aligned} \int_{\Omega \backslash B_{R\epsilon }(x_{0})}he^{4\pi\phi_{\epsilon }^{2}}\,dx\geq \int _{\Omega \backslash2B_{R\epsilon }(x_{0})} h\bigl(1+4\pi\phi_{\epsilon }^{2} \bigr)\,dx\geq\gamma+4\pi \frac { \Vert \sqrt{h} G \Vert ^{2}_{2}}{c^{2}}+o \biggl(\frac{1}{c^{2}} \biggr). \end{aligned}$$

Finally, we obtain

$$\begin{aligned} \int_{\Omega }he^{4\pi\phi_{\epsilon }^{2}}\,dx> \gamma+\pi h(x_{0}) e^{1+4\pi A_{0}}. \end{aligned}$$

This contradicts (2.11). Hence $c_{\epsilon }$ must be bounded and the elliptic estimates imply the existence of extremal functions. This completes the proof of Theorem 1.

## Proof of Theorem 1

Let *l* be a positive integer and $0\leq \alpha < \lambda _{l+1}(\Omega )$. Following the same steps as in the proof of Theorem 1, we see that, for any *ϵ*, $0<\epsilon <4\pi$, there exists some $u_{\epsilon }\in E_{l}^{\perp}\cap C^{1}(\overline{\Omega })$ with $\Vert u_{\epsilon }\Vert _{1,\alpha }=1$ such that

$$\begin{aligned} \int_{\Omega }h e^{(4\pi-\epsilon )u_{\epsilon }^{2}}\,dx=\sup_{u\in E_{l}^{\perp}, \Vert u \Vert _{1, \alpha }\leq1 } \int_{\Omega } he^{(4\pi-\epsilon )u^{2}}\,dx, \end{aligned}$$

where $\Vert \cdot \Vert _{1,\alpha }$ is defined as in (1.4). Moreover, the Euler–Lagrange equation for $u_{\epsilon }$ is

$$\begin{aligned} \textstyle\begin{cases}-\Delta u_{\epsilon}-\alpha u_{\epsilon }=\frac{1}{\lambda _{\epsilon}}hu_{\epsilon}e^{(4\pi-\epsilon ) u_{\epsilon}^{2}}\quad \text{in } \Omega ,\\ u_{\epsilon }\in E_{l}^{\perp}\cap C^{1}(\overline{\Omega }),\\ \lambda_{\epsilon}=\int_{\Omega } h u_{\epsilon}^{2}e^{(4\pi-\epsilon) u_{\epsilon}^{2}}\,dx. \end{cases}\displaystyle \end{aligned}$$

Let $c_{\epsilon }= \vert u_{\epsilon }(x_{\epsilon }) \vert =\max_{\overline{\Omega }} \vert u_{\epsilon }\vert $. We assume that $c_{\epsilon }\rightarrow\infty$ and $x_{\epsilon }\rightarrow x_{0}\in\overline{\Omega }$. Similar to (2.11), we obtain

3.1 $$ \sup_{u\in E_{l}^{\bot}, \Vert u \Vert _{1, \alpha }\leq1 } \int_{\Omega } he^{4\pi u^{2}}\,dx\leq \gamma+\pi h(x_{0})e^{1+4\pi A_{0}}, $$

where $\gamma=\int_{\Omega }h\,dx$.

Let $r(x)= \vert x-x_{0} \vert $. Define the same function

$$\begin{aligned} \phi_{\epsilon }(x)=\textstyle\begin{cases}c+\frac{1}{c} (-\frac{1}{4\pi}\log(1+\pi \frac {r^{2}}{\epsilon ^{2}})+B ) & \text{for } x\leq R\epsilon ,\\ \frac{G-\eta\Phi}{c}& \text{for } R\epsilon \leq r\leq2R\epsilon ,\\ \frac{G}{c}& \text{for } r>2R\epsilon , \end{cases}\displaystyle \end{aligned}$$

as in (2.12). Set

$$\begin{aligned} \widetilde{\phi}_{\epsilon }=\phi_{\epsilon }-\sum _{i=1}^{l}\sum_{j=1}^{n_{i}}( \phi _{\epsilon },e_{ij})e_{ij}, \end{aligned}$$

where $(e_{ij})\ (1\leq i\leq l, 1\leq j\leq n_{i})$ is the basis of $E_{l}$. Then, by (75) and (76) of [1], we have

$$\begin{aligned} \widetilde{\phi}_{\epsilon }=\phi_{\epsilon }+o \biggl( \frac{1}{\log^{2}\epsilon } \biggr) \end{aligned}$$

and

$$\begin{aligned} \Vert \widetilde{\phi}_{\epsilon }\Vert ^{2}_{1,\alpha }=1+o \biggl(\frac{1}{\log^{2}\epsilon } \biggr). \end{aligned}$$

Thus

$$\begin{aligned} \int_{\Omega } he^{4\pi\frac{\widetilde{\phi}_{\epsilon }^{2}}{ \Vert \widetilde {\phi }_{\epsilon }\Vert ^{2}_{1,\alpha }}}\,dx&= \int_{\Omega } he^{4\pi\phi_{\epsilon }^{2}+o (\frac {1}{\log \epsilon } )}\,dx \\ &\geq \biggl(1+o \biggl(\frac{1}{\log \epsilon } \biggr) \biggr) \biggl( \gamma+\pi h(x_{0}) e^{1+4\pi A_{0}} +4\pi\frac{ \Vert \sqrt{h} G \Vert ^{2}_{2}}{c^{2}}+o \biggl( \frac {1}{c^{2}} \biggr) \biggr) \\ &\geq \gamma+\pi h(x_{0}) e^{1+4\pi A_{0}} +4\pi\frac{ \Vert \sqrt{h} G \Vert ^{2}_{2}}{c^{2}}+o \biggl(\frac{1}{c^{2}} \biggr). \end{aligned}$$

Set $\widehat{\phi}_{\epsilon }=\frac{\widetilde{\phi}_{\epsilon }}{ \Vert \widetilde {\phi } \Vert _{1,\alpha }}$. Then $\int_{\Omega } he^{4\pi\widehat{\phi}_{\epsilon }^{2} }\,dx>\gamma +\pi h(x_{0}) e^{1+4\pi A_{0}}$. This contradicts (3.1). Hence $c_{\epsilon }$ must be bounded and the extremal function exists. We finish the proof of Theorem 2.

## Proof of Theorem 3

First, we prove that, for any $0<\epsilon <4\pi$, there exists some $u_{\epsilon }\in C^{1}(\Sigma )$ such that

4.1 $$ \int_{\Sigma}he^{(4\pi-\epsilon ) u_{\epsilon }^{2}}\,dv_{g}=\sup _{u\in W^{1,2}(\Sigma ),\int_{\Sigma }u \,dv_{g}=0, \Vert u \Vert _{1,\alpha }\leq1} \int_{\Sigma }he^{(4\pi-\epsilon ) u^{2}}\,dv_{g} $$

with $\Vert u_{\epsilon }\Vert _{1,\alpha }=1$ and $\int_{\Sigma}u_{\epsilon }\,dv_{g}=0$.

The main procedure of the proof is as follows. Since $0\leq \alpha <\lambda _{1}(\Sigma )$, we may choose a bounded sequence $u_{j}$ in $W^{1,2}(\Sigma )$ such that

$$\begin{aligned} \int_{\Sigma}he^{(4\pi-\epsilon )u_{j}^{2}}\,dv_{g}\rightarrow\sup _{u\in W^{1,2}(\Sigma ),\int_{\Sigma }u \,dv_{g}=0, \Vert u \Vert _{1,\alpha }\leq1} \int_{\Sigma }he^{(4\pi-\epsilon )u^{2}}\,dv_{g}. \end{aligned}$$

There exists some $u_{\epsilon }\in W^{1,2}(\Sigma )$ such that up to a subsequence,

$$\begin{aligned} &u_{j}\rightharpoonup u_{\epsilon } \quad\text{weakly in } W^{1,2}(\Sigma ), \\ &u_{j}\rightarrow u_{\epsilon } \quad \text{strongly in } L^{2}(\Sigma ), \\ &u_{j}\rightarrow u_{\epsilon } \quad \text{a.e. in }\Sigma . \end{aligned}$$

Using the same argument as in the proof of Theorem 3 in [1], we get $he^{(4\pi-\epsilon )u_{j}^{2}}$ is bounded in $L^{q}$ for some $q>1$. Hence $he^{(4\pi-\epsilon )u_{j}^{2}}\rightarrow he^{(4\pi-\epsilon )u_{\epsilon }^{2}}$ strongly in $L^{1}(\Sigma )$. Hence (4.1) holds. The fact that $\int_{\Sigma }u_{j}\,dv_{g}=0$ implies $\int_{\Sigma }u_{\epsilon }\,dv_{g}=0$. We also have $\Vert u_{\epsilon }\Vert _{1,\alpha }=1$ by contradiction as in the proof of Lemma 6.

Moreover, $u_{\epsilon }$ satisfies the Euler–Lagrange equation

$$\begin{aligned} \textstyle\begin{cases}\Delta_{g} u_{\epsilon}-\alpha u_{\epsilon }=\frac{1}{\lambda _{\epsilon}}hu_{\epsilon}e^{(4\pi-\epsilon ) u_{\epsilon}^{2}}-\frac{\mu_{\epsilon }}{\lambda _{\epsilon }},\\ \lambda_{\epsilon}=\int_{\Sigma } h u_{\epsilon}^{2}e^{(4\pi-\epsilon) u_{\epsilon}^{2}}\,dv_{g},\\ \mu_{\epsilon }=\frac{1}{{\mathrm{ Vol}_{g}}(\Sigma )}\int_{\Sigma}h u_{\epsilon }e^{(4\pi -\epsilon )u_{\epsilon }^{2}}\,dv_{g}, \end{cases}\displaystyle \end{aligned}$$

where $\Delta _{g}$ denotes the Laplace–Beltrami operator.

Denote $c_{\epsilon }= \vert u_{\epsilon }(x_{\epsilon }) \vert =\max_{\Sigma } \vert u_{\epsilon }\vert $. If $c_{\epsilon }$ is bounded, the existence of the extremal function follows from the elliptic estimates. We assume that $c_{\epsilon }\rightarrow+\infty$ and $x_{\epsilon }\rightarrow p\in \Sigma $. Similar to Lemma 7, we have $h(p)>0$. Choosing an isothermal coordinate system $(U,\phi)$ near *p* such that the metric *g* can be written as $g=e^{f}(dx_{1}^{2}+dx_{2}^{2})$, where $f\in C^{1}(\phi(U), \mathbb {R})$ and $f(0)=0$. Denote $\Omega =\phi(U)$, $\widetilde{u}_{\epsilon }=u_{\epsilon }\circ \phi^{-1}$ and $\widetilde{x}_{\epsilon }=\phi(x_{\epsilon })$. Let

$$\begin{aligned} r_{\epsilon }=\sqrt{ \lambda _{\epsilon }}\bigl[h(p)\bigr]^{-1/2}c_{\epsilon }^{-1}e^{-(2\pi-\epsilon /2)c_{\epsilon }^{2}}. \end{aligned}$$

Define

$$\begin{aligned} \psi_{\epsilon }(x)=c_{\epsilon }^{-1}\widetilde{u}_{\epsilon }( \widetilde{x}_{\epsilon }+r_{\epsilon }x) \end{aligned}$$

and

$$\begin{aligned} \varphi_{\epsilon }(x)=c_{\epsilon }\bigl(\widetilde{u}( \widetilde{x}_{\epsilon }+r_{\epsilon }x)-c_{\epsilon }\bigr) \end{aligned}$$

for $x\in \Omega _{\epsilon }=\{x\in\mathbb{R}^{2}: \widetilde{x}_{\epsilon }+r_{\epsilon }x\in \Omega \}$. Then we get

$$\begin{aligned} &{-}\Delta _{\mathbb{R}^{2}}\psi_{\epsilon }=e^{f(\widetilde{x}_{\epsilon }+r_{\epsilon }x)} \biggl(\alpha r_{\epsilon }^{2}\psi_{\epsilon }+\frac{h\psi_{\epsilon }e^{(4\pi-\epsilon )(\widetilde{u}_{\epsilon }^{2}-c_{\epsilon }^{2})}}{c_{\epsilon }^{2} h(p)}- \frac{\mu_{\epsilon }}{c_{\epsilon }^{3} e^{(4\pi-\epsilon )c_{\epsilon }^{2}}h(p)} \biggr), \\ &{-}\Delta _{\mathbb{R}^{2}}\varphi_{\epsilon }=e^{f(\widetilde{x}_{\epsilon }+r_{\epsilon }x)} \biggl(\alpha r_{\epsilon }^{2} c_{\epsilon }^{2} \psi_{\epsilon }+\frac{h\psi_{\epsilon }e^{(4\pi-\epsilon )(1+\psi _{\epsilon })\varphi _{\epsilon }}}{ h(p)}-\frac{\mu_{\epsilon }}{c_{\epsilon }e^{(4\pi-\epsilon )c_{\epsilon }^{2}}h(p)} \biggr), \end{aligned}$$

where $-\Delta _{\mathbb{R}^{2}}$ is the usual Laplace operator in $\mathbb {R}^{2}$. By the same argument as in Sect. 2.2, we obtain

$$\begin{aligned} \psi_{\epsilon }\rightarrow1 \quad\text{in } C^{1}_{\mathrm{loc}} \bigl(\mathbb{R}^{2}\bigr) \end{aligned}$$

and

$$\begin{aligned} \varphi_{\epsilon }\rightarrow\varphi\quad\text{in } C^{1}_{\mathrm{loc}} \bigl(\mathbb{R}^{2}\bigr), \end{aligned}$$

where

$$\begin{aligned} \varphi(x)=-\frac{1}{4\pi}\log \bigl(1+\pi \vert x \vert ^{2} \bigr) \end{aligned}$$

and

$$\begin{aligned} \int_{\mathbb{R}^{2}} e^{8\pi\varphi}\,dx=1. \end{aligned}$$

We also have $c_{\epsilon }u_{\epsilon }\rightharpoonup G$ weakly in $W^{1,q}(\Sigma )$ for all $1< q<2$, and $c_{\epsilon }u_{\epsilon }\rightarrow G$ in $C^{1}_{\mathrm{loc}}(\Sigma \backslash\{p\})\cap L^{2}(\Sigma )$, where *G* is Green function satisfying

$$\begin{aligned} \Delta _{g} G-\alpha G=\delta_{p}-\frac{1}{\mathrm{Vol}_{g}(\Sigma )}\quad \text{in } \Sigma \end{aligned}$$

and $\int_{\Sigma}G\,dv_{g}=0$. As before, *G* can be represented by

$$\begin{aligned} G=-\frac{1}{2\pi}\log r+A_{p} +\Phi_{p}, \end{aligned}$$

where *r* is the geodesic distance from *p*, $A_{p}$ is a constant and $\Phi_{p}\in C^{1}(\Sigma )$ with $\Phi_{p}(p)=0$.

Similar to (2.11), we can get

4.2 $$ \sup_{u\in W^{1,2}(\Sigma ),\int_{\Sigma }u \,dv_{g}=0, \Vert u \Vert _{1,\alpha }\leq 1} \int _{\Sigma }he^{4\pi u^{2}}\,dv_{g}\leq \gamma _{1}+\pi h(p) e^{1+4\pi A_{p}}, $$

where $\gamma _{1}=\int_{\Sigma}h\,dv_{g}$.

For the extremal function, define

4.3 $$ \phi_{\epsilon }(x)=\textstyle\begin{cases}c+\frac{1}{c} (-\frac{1}{4\pi}\log(1+\pi \frac {r^{2}}{\epsilon ^{2}})+B ) & \text{for } x\leq R\epsilon ,\\ \frac{G-\eta\Phi_{p}}{c}& \text{for } R\epsilon \leq r\leq2R\epsilon ,\\ \frac{G}{c}& \text{for } r>2R\epsilon , \end{cases} $$

as in [1], where *c* and *B* are constants, $R=-\log \epsilon $, $\eta\in C^{\infty}_{0}(B_{2R\epsilon }(p))$ with $\eta=1$ on $B_{R\epsilon }(p)$ and $\Vert \nabla _{g} \eta \Vert _{L^{\infty}}=O(\frac{1}{R\epsilon })$. Choose

$$\begin{aligned} &c= \biggl(\frac{-\log \epsilon -2\pi B+2\pi A_{p}+\frac{1}{2}\log\pi+O (\frac {1}{R^{2}} )}{2\pi} \biggr)^{1/2}, \\ &B=\frac{1}{4\pi}+O \biggl( \frac{1}{R^{2}} \biggr) +O \bigl(R\epsilon \log(R\epsilon ) \bigr), \end{aligned}$$

as in [1] such that $\phi_{\epsilon }\in W^{1,2}(\Sigma )$ and $\Vert \phi _{\epsilon }-\bar{\phi}_{\epsilon } \Vert _{1,\alpha }=1$, where

$$\begin{aligned} \overline{\phi}_{\epsilon }=\frac{1}{\mathrm{ Vol}_{g}(\Sigma )} \int_{\Sigma}\phi _{\epsilon }\,dv_{g} . \end{aligned}$$

Then we have on $B_{R\epsilon }(p)$

$$\begin{aligned} 4\pi (\phi_{\epsilon }-\overline{\phi}_{\epsilon })^{2}\geq4 \pi c^{2}-2\log \biggl(1+\pi\frac {r^{2}}{\epsilon ^{2}} \biggr)+8\pi B+O \bigl(R \epsilon \log(R\epsilon ) \bigr). \end{aligned}$$

It follows that

4.4 $$ \int_{B_{R\epsilon }(p)}h e^{4\pi(\phi_{\epsilon }-\overline{\phi}_{\epsilon })^{2}}\,dv_{g}\geq \pi h(p) e^{1+4\pi A_{p}} +O \biggl(\frac{1}{(\log \epsilon )^{2}} \biggr) $$

and

4.5 $$\begin{aligned} \int_{\Sigma \backslash B_{R\epsilon }(p)} he^{4\pi(\phi_{\epsilon }-\overline {\phi}_{\epsilon })^{2}}\,dv_{g} &\geq \int_{\Sigma \backslash B_{2R\epsilon }(p)}\bigl(1+4\pi\phi_{\epsilon }^{2}\bigr) \,dv_{g} +O \bigl(R\epsilon \log(R\epsilon ) \bigr) \\ &\geq \gamma _{1}+4\pi\frac{ \Vert \sqrt{h} G \Vert ^{2}_{2}}{c^{2}}+o \biggl(\frac {1}{c^{2}} \biggr). \end{aligned}$$

Combining (4.4) and (4.5), we find a contradiction with (4.2). Hence $c_{\epsilon }$ must be bounded. Using the elliptic estimates, we have the existence of the extremal function.

## Proof of Theorem 5

Let *l* be a positive integer and $0\leq \alpha <\lambda _{l+1}(\Sigma )$. First, by the same arguments, we obtain for any *ϵ*, $0<\epsilon <4 \pi$, there exists some $u_{\epsilon }\in E^{\perp}_{l}\cap C^{1}(\Sigma )$ satisfying $\Vert u_{\epsilon }\Vert _{1,\alpha }=1$ and

5.1 $$ \int_{\Sigma}he^{(4\pi-\epsilon )u_{\epsilon }^{2}}\,dv_{g}= \sup _{u\in E^{\perp}_{l}, \Vert u \Vert _{1,\alpha }\leq 1} \int_{\Sigma }he^{(4\pi-\epsilon )u^{2}}\,dv_{g}. $$

Moreover, $u_{\epsilon }$ satisfies the Euler–Lagrange equation

$$\begin{aligned} \textstyle\begin{cases}\Delta_{g} u_{\epsilon}-\alpha u_{\epsilon }=\frac{1}{\lambda _{\epsilon}}hu_{\epsilon}e^{(4\pi-\epsilon ) u_{\epsilon}^{2}}-\frac{\mu_{\epsilon }}{\lambda _{\epsilon }},\\ u_{\epsilon }\in E^{\perp}_{l}\cap C^{1}(\Sigma ),\\ \lambda_{\epsilon}=\int_{\Sigma } h u_{\epsilon}^{2}e^{(4\pi-\epsilon) u_{\epsilon}^{2}}\,dv_{g},\\ \mu_{\epsilon }=\frac{1}{{\mathrm{ Vol}_{g}}(\Sigma )}\int_{\Sigma}h u_{\epsilon }e^{(4\pi -\epsilon )u_{\epsilon }^{2}}\,dv_{g}. \end{cases}\displaystyle \end{aligned}$$

Let $c_{\epsilon }=\max_{\Sigma } \vert u_{\epsilon }\vert $. We assume that $c_{\epsilon }= \vert u_{\epsilon }(x_{\epsilon }) \vert \rightarrow +\infty$ and $x_{\epsilon }\rightarrow p\in \Sigma $. We also get the upper bound estimate

5.2 $$ \sup_{u\in E^{\perp}_{l},\int_{\Sigma }u \,dv_{g}=0, \Vert u \Vert _{1,\alpha }\leq1} \int _{\Sigma }he^{4\pi u^{2}}\,dv_{g}\leq \gamma _{1}+\pi h(p) e^{1+4\pi A_{p}}, $$

where $\gamma _{1}=\int_{\Sigma}h\,dv_{g}$.

For the existence of the extremal function, we define $\phi_{\epsilon }$ as in (4.3). Then we have

$$\begin{aligned} \int_{\Sigma } he^{4\pi(\phi_{\epsilon }-\overline{\phi}_{\epsilon })^{2}}\,dv_{g}\geq \gamma _{1}+\pi h(p) e^{1+4\pi A_{p}}+4\pi\frac{ \Vert \sqrt{h} G \Vert ^{2}_{2}}{c^{2}}+o \biggl( \frac {1}{c^{2}} \biggr). \end{aligned}$$

Set

$$\begin{aligned} \widetilde{\phi}_{\epsilon }=\phi_{\epsilon }-\overline{ \phi}_{\epsilon }-\sum_{i=1}^{l}\sum _{j=1}^{n_{i}}(\phi_{\epsilon }-\overline{ \phi}_{\epsilon },e_{ij})e_{ij}, \end{aligned}$$

where

$$\begin{aligned} \overline{\phi}_{\epsilon }=\frac{1}{\mathrm{ Vol}_{g}(\Sigma )} \int_{\Sigma}\phi _{\epsilon }\,dv_{g} \end{aligned}$$

and $(e_{ij})\ (1\leq i\leq l, 1\leq j\leq n_{i})$ is the basis of $E_{l}$.

Then, by (102) and (103) in [1], we have

$$\begin{aligned} \widetilde{\phi}_{\epsilon }=\phi_{\epsilon }-\overline{ \phi}_{\epsilon }+o \biggl(\frac {1}{\log^{2}\epsilon } \biggr) \end{aligned}$$

and

$$\begin{aligned} \Vert \widetilde{\phi}_{\epsilon }\Vert ^{2}_{1,\alpha }=1+o \biggl(\frac{1}{\log^{2}\epsilon } \biggr). \end{aligned}$$

Thus

$$\begin{aligned} \int_{\Sigma}he^{4\pi\frac{\widetilde{\phi}_{\epsilon }^{2}}{ \Vert \widetilde {\phi}_{\epsilon }\Vert ^{2}_{1,\alpha }}}\,dv_{g}&= \int_{\Sigma}he^{4\pi(\phi_{\epsilon }-\overline {\phi}_{\epsilon })^{2}+o (\frac{1}{\log \epsilon } )}\,dv_{g} \\ &\geq \biggl(1+o \biggl(\frac{1}{\log \epsilon } \biggr) \biggr) \biggl( \gamma_{1}+\pi h(p) e^{1+4\pi A_{p}} +4\pi\frac{ \Vert \sqrt{h} G \Vert ^{2}_{2}}{c^{2}}+o \biggl( \frac {1}{c^{2}} \biggr) \biggr) \\ &\geq \gamma_{1}+\pi h(p) e^{1+4\pi A_{p}} +4\pi\frac{ \Vert \sqrt{h} G \Vert ^{2}_{2}}{c^{2}}+o \biggl(\frac{1}{c^{2}} \biggr). \end{aligned}$$

Set $\widehat{\phi}_{\epsilon }=\frac{\widetilde{\phi}_{\epsilon }}{ \Vert \widetilde {\phi}_{\epsilon }\Vert _{1,\alpha }}$. We have

$$\begin{aligned} \int_{\Sigma}he^{\widehat{\phi}_{\epsilon }^{2}}\,dv_{g}> \gamma_{1}+\pi h(p) e^{1+4\pi A_{p}}. \end{aligned}$$

This contradicts (5.2). Hence $c_{\epsilon }$ must be bounded and the extremal function exists. We finish the proof.
